# Natural and man-made V-gene repertoires for antibody discovery

**DOI:** 10.3389/fimmu.2012.00342

**Published:** 2012-11-15

**Authors:** William J. J. Finlay, Juan C. Almagro

**Affiliations:** ^1^Global Biotherapeutics Technologies, PfizerDublin, Ireland; ^2^Centers for Therapeutic Innovation, PfizerBoston, MA, USA

**Keywords:** therapeutic antibodies, antigen-binding site, antibody structure, structure-function relationship

## Abstract

Antibodies are the fastest-growing segment of the biologics market. The success of antibody-based drugs resides in their exquisite specificity, high potency, stability, solubility, safety, and relatively inexpensive manufacturing process in comparison with other biologics. We outline here the structural studies and fundamental principles that define how antibodies interact with diverse targets. We also describe the antibody repertoires and affinity maturation mechanisms of humans, mice, and chickens, plus the use of novel single-domain antibodies in camelids and sharks. These species all utilize diverse evolutionary solutions to generate specific and high affinity antibodies and illustrate the plasticity of natural antibody repertoires. In addition, we discuss the multiple variations of man-made antibody repertoires designed and validated in the last two decades, which have served as tools to explore how the size, diversity, and composition of a repertoire impact the antibody discovery process.

## Introduction

In recent decades, rodent monoclonal antibodies obtained by hybridoma technology and engineered by molecular biology techniques, or human antibodies obtained by display technologies or B-cell cloning, have become the treatment of choice in diverse diseases such as multiple sclerosis, rheumatoid arthritis, and several types of cancers, making a significant component of the pharmaceuticals market (Nelson et al., 2010). The success of therapeutic antibodies, with as many as 28 antibodies and antibody fragments marketed in The United States or The European Union (Reichert, 2012), resides in their exquisite specificity, high potency, stability, solubility, clinical tolerability, and relatively inexpensive manufacturing process in comparison with other biologics.

The factors contributing to the specificity and potency of antibodies have intrigued scientists since their discovery in the late 1800s and only in the last three decades has a clear picture of how antibodies work emerged. The current knowledge base has been assembled by combining insights from multiple disciplines such as: structural biology—studying hundreds of x-ray crystallography antibody structures from different species (Davies and Metzger, 1983; Chothia and Lesk, 1987; Wilson and Stanfield, 1994; Stanfield and Wilson, 2010) free and in complex with a wide variety of ligands (MacCallum et al., 1996; Ragunathan et al., 2012); immunogenetics—by fully characterizing the germline gene antibody repertoire of humans and other species (Lefranc et al., 2005) and by deciphering the molecular mechanisms used to generate functional antibody molecules starting from diverse gene families (Tonegawa, 1983); and cellular immunology—dissecting the process by which *in vivo* selection of specific antibodies occurs during an immune response and understanding the mechanisms that allow the affinity and specificity of the selected antibodies to mature as the immune response progresses (Noia and Neuberger, 2007).

The accumulation of this knowledge has potentiated several technological advances in the antibody engineering field, such as humanization of non-human antibodies to increase their human content and to enhance their manufacturability profile (Gilliland et al., 2012), the development of display technologies to select specific human antibodies *in vitro* (Hoogenboom, 2005), and the engineering of antibody characteristics such as affinity, cross-reactivity with target orthologs, stability, and solubility. Each of these great leaps forward have relied directly on a core of fundamental immunological knowledge and made it possible to create close to 30 antibody-based drugs, at the time of writing.

Here, we first provide an overview of the antibody structure and outline the fundamental principles that define how antibodies interact with diverse ligands. In the second section, we review the current knowledge of the antibody repertoire of humans and experimental species commonly used to generate monoclonal antibodies such as mice, chickens, and camelids. Each of these species possess distinct germline gene repertoires, have differing mechanisms of generating and affinity maturing their antibody molecules and, therefore, offer alternative sources of specific variable regions for therapeutic antibody development. In the third section, multiple variations of man-made antibody repertoires are described, from their inception to the current state of the art. These designer repertoires have applied the compound knowledge derived from both structural and repertoire studies, serving as tools to test hypotheses on how the size of a repertoire, its diversity and composition impact the selection of more specific and higher affinity antibodies. These repertoires have also been used extensively by academic laboratories and biotech companies to discover and optimize human antibodies *in vitro*. At the end of the article, a section with conclusions and future directions is included.

## The antibody molecule

The IgG isotype is the most abundant form of circulating antibody and the molecular format of choice for most marketed therapeutic antibodies (Reichert, 2012), as it is stable, soluble, readily expressed in heterologous systems such as Chinese hamster ovary (CHO) cells and can potentially engage effector functions such as antibody-dependent cell-mediated cytotoxicity (ADCC) and complement-dependent cytotoxicity (CDC). IgGs are Y-shaped glycoproteins of approximately 150 kDa composed of two identical polypeptide heavy (H) chains and two identical light (L) chains. The most abundant classes of L chains are κ and λ, which are functionally indistinguishable, but structurally different and vary in proportion in different species. For instance, the human repertoire is approximately 40:60 λ:κ, whereas, the mouse repertoire is ~95% κ-type. The H chain divides Igs into five classes, IgG, IgD, IgE, IgA, and IgM, each with a unique role in the adaptive immune system.

**Figure 1:**
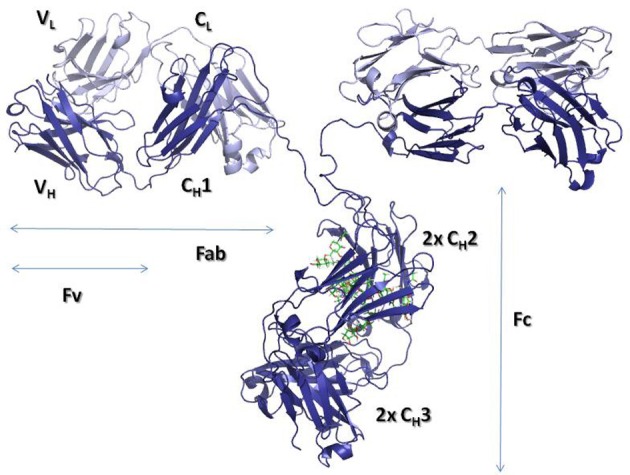
**Ribbon representation of an intact IgG molecule (PDBID: 1IGT).** The heavy chains are shown in dark blue, while the Light chains are colored in light blue. The carbohydrate moieties attached to the C_H_2 domains are represented with sticks. The figure was produced using PyMol (DeLano, 2002. *The PyMOL molecular graphics system*. Delano Scientific, San Carlos, CA).

By digesting IgGs with papain, two fractions can be obtained, one containing the so-called crystallizable fragment (Fc) and the other containing two identical antigen-binding fragments or Fabs (Figure 1). In the Fc resides the effector functions, whereas, the Fab, as its name indicates, binds the antigen and thus defines the specificity of antibodies. Each Fab has two variable domains, one from the H chain (V_H_) and another from the L chain (V_L_), in addition to two C domains: C_H_1 and C_L_. The Fc is a dimer made of four C domains, two C_H_2 and two C_H_3 domains.

**Figure 2:**
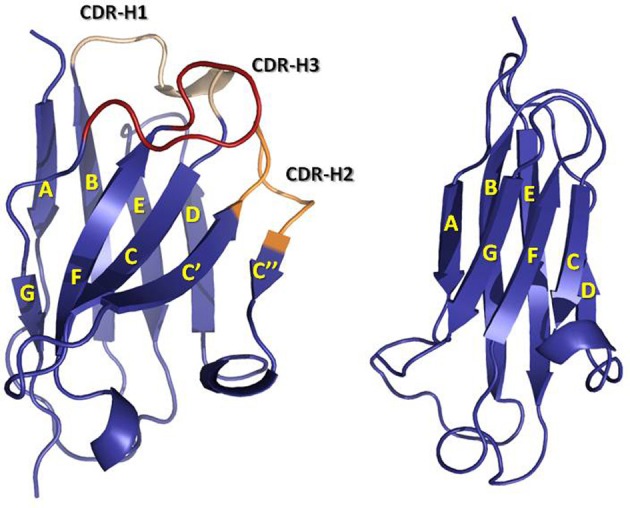
**Ribbon representation of a V_H_ (left) domain and a C_H_1 (right) domain.** CDRs are colored in yellow (CDR-1), orange (CDR-2), and red (CDR-3). Note the insertion in the V_H_ domain with respect to the C_H_1 domain of two β-strands, C' and C”, and the loop linking them, which contains the CDR-H2. The coordinates used to produce the Figure were the same as in Figure 1. The figure was generated with PyMol.

The first antibody structures, solved in the 1970s [for early reviews see (Padlan, 1977; Amzel and Poljak, 1979; Davies and Metzger, 1983) and for more current reviews see (Wilson and Stanfield, 1994; Stanfield and Wilson, 2010)], revealed that V- and C-domains have a conserved and similar structure, termed “immunoglobulin (Ig) fold.” The Ig fold is also the building block of a large number of other proteins with diverse functions, which are collectively called the Ig superfamily (Williams and Barclay, 1988). The Ig fold consists of two anti-parallel β-sheets that are tightly packed together. In the C domain, one of the β-sheets is formed by four β-strands A to D, whereas, the other β-sheet is formed by three β-strands C to G (Figure 2). A conserved intra-domain disulfide bridge, formed between cysteine residues in the B and F β-strands, stabilizes the C domain. The V-domain have an insertion with respect to the C domain of two extra β-strands, identified as C' and C”, present between β-strands C and D (Figure 2). As in the C domain, an intra-domain disulfide bridge is formed between cysteine residues in β-strands B and F. The V-domains are in general less compact than the C domains with some longer loops connecting the β-strands. This flexibility and the longer loops contribute to the mechanism of antigen binding, thus defining the capability of antibodies to recognize diverse antigens.

## The antigen-binding site

**Figure 3:**
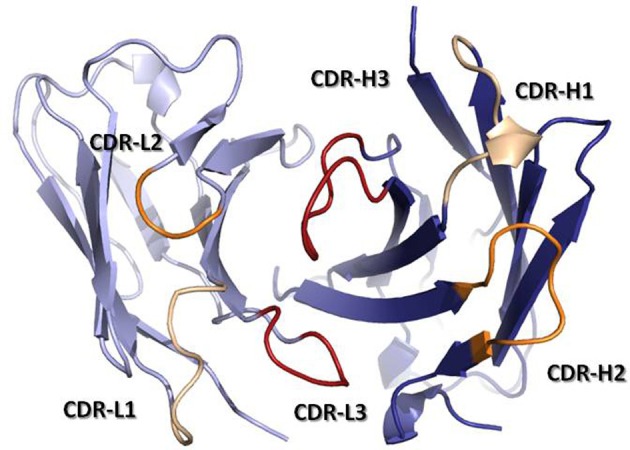
**Ribbon representation of a Fv fragment seen from the antigen perspective.** V_H_ is colored in dark blue, while V_L_ is colored in light blue. CDRs are colored in yellow (CDR-1), orange (CDR-2), and red (CDR-3). The coordinates used to produce the Figure are the same as in Figure 1. The Figure was generated with PyMol.

The antigen binding site is principally defined by the Complementarity-Determining Regions (CDRs). These regions were originally identified by amino acid sequence variability analysis (Wu and Kabat, 1970; Kabat and Wu, 1971) as highly variable regions within the V-domains. The CDRs were defined prior to our knowledge of the mechanisms by which antibodies are generated and predated the three-dimensional structure solution of antibodies. Once the first Fab structures were solved, it was realized that the CDRs approximately correspond to loops that vary in structure, called hypervariable loops (HVLs). Each V-domain contributes three CDRs to the antigen-binding site: CDR-L1, CDR-L2, and CDR-L3 from the V_L_ and CDR-H1, CDR-H2, and CDR-H3 from the V_H_. The three CDRs from V_H_ and the three from V_L_ are brought together by non-covalent association of the V-domains at the N-terminal region of the Fv (Figure 3). The remaining portion of the V-domain, i.e., the two β-sheets and non-HVLs, generally provide structural support to the antigen-binding site, rather than making contact with antigen and are thus referred to as framework regions (FRs). However, the sequence variability observed in the FRs is not irrelevant to functional binding diversity, as it can directly affect CDR loop conformation and the orientation of V_H_–V_L_ pairing (Foote and Winter, 1992; Abhinandan and Martin, 2010).

Given the essential variability of the antigen-binding site, which must be capable of recognizing a large array of diverse antigens to fulfill its remit, it was initially thought that each antibody possesses a unique conformation at the antigen-binding site. Nevertheless, analysis (Chothia and Lesk, 1987; Chothia et al., 1989) in the late 1980s of a small set of structures of immunoglobulin fragments available at the time revealed that, although the HVLs vary in sequence, five out of the six HVLs (CDR-L1, CDR-L2, CDR-L3, CDR-H1, and CDR-H2) had a limited set of main-chain conformations or “canonical structures.” The canonical structure model implied a paradigm shift in the field, replacing the notion that each antibody has unique HVL conformations and thus overall unique antigen-binding site structure. The limited set of canonical structures helped to develop 3D modeling structure strategies (Martin and Thornton, 1996) and suggested that structural constraints are at work in antigen recognition.

A canonical structure is defined by the HVL length and conserved residues located in the HVL and FR (Chothia and Lesk, 1987). Overall, the structural repertoire generated by λ-type chains is broader than that of κ-type chains (Chailyan et al., 2011). In the latter, all the canonical structures at CDR-L1 follow a similar pattern, which consists of an extended conformation between residues 26 and 29 [Chothia's numbering; (Al-Lazikani et al., 1997)], and hairpin loops of different lengths encompassing residues 30–32, with up to seven insertions in this segment of the loop. The CDR-L2 adopts a single conformation. Most (~70%) of the CDR-L3 loops have a single canonical structure. In contrast, the CDR-L1 of λ-type chains adopts a helical structure with up to eight conformations and an average root mean square deviation (RMSD) between loops of 2.3 Å (Chailyan et al., 2011). The λ-type CDR-L2 usually adopts a similar hairpin loop conformation to that of κ-type, but can in some instances have an insertion of four residues, which leads to another canonical structure. The CDR-L3 in λ-type antibodies has a broader variety of lengths and conformations than κ-type antibodies, with only a small fraction of the loops following a defined canonical structure (Chailyan et al., 2011).

The CDR-H1, similar to the CDR-L1, has an extended conformation linking β-strands from the two β-sheets that form the Ig fold. However, it is less diverse than its counterpart in V_L_, with three canonical structures and a strong bias (~85%) (Ragunathan et al., 2012) toward the shortest loop (seven residues). The repertoire of canonical structures of CDR-H2 is less skewed than that for CDR-L3 and CDR-H1, with six canonical structures. Still, 59–70% of the antibodies (Ragunathan et al., 2012) have a six-residue canonical structure.

Recently, the application of clustering algorithms (North et al., 2011) on 300 non-redundant antibody structures has further stratified the canonical structure combinations by identifying 28 HVL combinations of lengths for the loops with canonical structures, whereas, previous analysis (Al-Lazikani et al., 1997) covered only 20. Only four of these clusters had more than one conformation, of which two could be distinguished by gene source (mouse/human; κ/λ) and one could be distinguished solely by the presence and position of Proresidues in the CDR-L3. Of the 28 CDR-lengths, 15 have multiple conformational clusters, including 10 for which previous analysis had only one canonical structure combination.

The CDR-H3, localized at the center of the antigen-binding site, is by far the most variable loop in length and sequence of the CDRs (Chothia and Lesk, 1987; Wu et al., 1993; Zemlin et al., 2003). The diversity of the CDR-H3 comes from the recombination of three germline genes: IGHV, IGHD, and IGHJ (Tonegawa, 1983), imprecise recombination of these genes, i.e., junctional diversity (Alt and Baltimore, 1982), the possibility of using three reading frames for translation of the IGHD gene (Sanz, 1991), and further diversification during somatic hypermutation process (see below).

Human CDR-H3 loops have an average length of 15.2 (±4.1) residues (IMGT CDR definition) (Zemlin et al., 2003), with a range of lengths between 1–35 residues, and a length distribution resembling a Gaussian process. While extensive analysis of antibody structures has identified sequence patterns to predict the conformation of the residues at the base of the CDR-H3 (Shirai et al., 1996; Morea et al., 1998), the enormous variability in amino acid sequence and length of this loop, as well as its flexibility, has precluded delineation of rules for predicting its overall conformation. Thus, structural modeling of CDR-H3 is still challenging (Almagro et al., 2011), using either comparative methods that rely on templates chosen based on sequence homology, or knowledge-based methods such as the canonical structure model.

## Structure-function relationships at the antigen-binding site

Since antibodies have a small subset of canonical structures in five of the six loops that define the antigen-binding site, it is reasonable to hypothesize that only a limited subset of antigen-binding site geometries exists, and the arising questions are whether the general architecture of the antigen-binding site can be predicted and whether it correlates with antigen recognition (Vargas-Madrazo et al., 1995). Finding structure-function correlations at the antigen-biding site holds the promise of providing insights into the mechanism of the molecular recognition process used by antibodies to bind diverse antigens and thereby to assist the rational design of antibodies of desired specificity.

Initial work (Vargas-Madrazo et al., 1995) showed that from a total of 300 possible canonical structure combinations described at that time, only 10 exist in 90% of the sequences analyzed. The existing canonical structure combinations were classified in two sets: one with preference for some specific types of antigens like proteins, peptides or haptens, and other with multi-specific binding capabilities. In the specific classes, the length of CDR-H2 and CDR-L1 was found to correlate with the type of antigen, whereas, in the multi-specific classes, such a correlation could not be established. A recent study (Ragunathan et al., 2012) of 140 unique antigen-antibody complexes has corroborated that most of the anti-protein antibodies have canonical structures determined by short CDR-L1 loops (6–8 residues). This is in contrast to anti-peptide and anti-hapten antibodies, which predominantly have canonical structures made of long CDR-L1 loops (11–13 residues). The remaining loops show little difference in the canonical structure distribution across anti-protein, anti-peptide, and anti-hapten antibodies.

**Figure 4:**
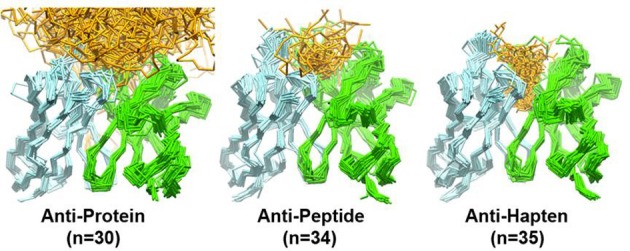
**Trace representation of 99 unique mid to high resolution (= 3.0 Å) Fv structures, including 30 in complex with proteins, 34 with peptides, and 35 with haptens.** V_H_ colored in dark blue. V_L_ colored in light blue. Ligands are colored in orange. The coordinates used to generate the Figure are listed in Ragunathan et al. (2012). The structures were superposed in Discovery Studio using the FR Cα atoms.

Figure 4 overlays 99 unique mid to high resolution (≤3.0 Å) antibody structures, including 30 in complex with proteins, 34 with peptides, and 35 with haptens. As can be seen, the topography of the antigen-binding site tends to determine the size of the antigen with which the antibody interacts. Anti-protein antibodies tend to have flatter binding sites than anti-peptide antibodies. The antigen-binding of anti-peptide antibodies is grooved, mainly determined by the long CDR-L1, which accommodates the peptides at the center of the antigen-binding site. Anti-hapten antibodies have a smaller antigen-biding site with contacts with haptens being buried deeper in the V_H_:V_L_ interface where proteins and peptides cannot reach.

In non-specific classes, the CDR-H3 plays a predominant role in defining the topography of the binding site (Vargas-Madrazo et al., 1995). Short CDR-H3 loops can create a cavity in the antigen-binding site to accommodate peptides. Long CDR-H3 loops are found in antibodies associated with chronic viral infections, in contrast to antibodies from acute viral infections, which have relatively short CDR-H3 loops (Breden et al., 2011). Long and extended CDR-H3 loops can generate a definite type of structure, called finger-like topography (Saphire et al., 2001), which differ from the typical flat anti-protein binding-site. This finger-like topography allows antibodies to access recessed epitopes in the viral proteins.

The number of residues in contact with antigens also differs in antibodies recognizing proteins, peptides and haptens (MacCallum et al., 1996; Almagro, 2004; Ragunathan et al., 2012). The average number of contact residues in V_L_ for the anti-protein, anti-peptide, and anti-hapten antibodies is 9, 9, and 7, respectively (Ragunathan et al., 2012). The corresponding values for V_H_ are 14, 12, and 10. A more detailed analysis of the solvent accessible surface (SAS) that is buried upon antigen binding and the location and frequency of contacts (called specificity-determining residues usage, SDRUs) of antibodies in complex with proteins, peptides or haptens also show distinctive patterns (Almagro, 2004). Anti-protein antibodies have an average (±SD) SAS value of 737 (±272) Å^2^ with hotspots of SDRUs located at the edge of the antigen-binding site (Ragunathan et al., 2012). Anti-hapten antibodies have a roughly 2-fold smaller SAS value of 374 (±117) Å^2^ with hotspots of SDRUs placed in the interior of the antigen-binding site or even buried in the V_L_:V_H_ interface. Anti-peptide antibodies have a SAS value of 544 (±158) Å^2^, which is in between anti-protein and anti-hapten antibodies. The SDRU hotspots of anti-peptide antibodies are located in the interior of the antigen-binding site but not buried in the V_L_:V_H_ interface as with anti-hapten antibodies.

**Figure 5:**
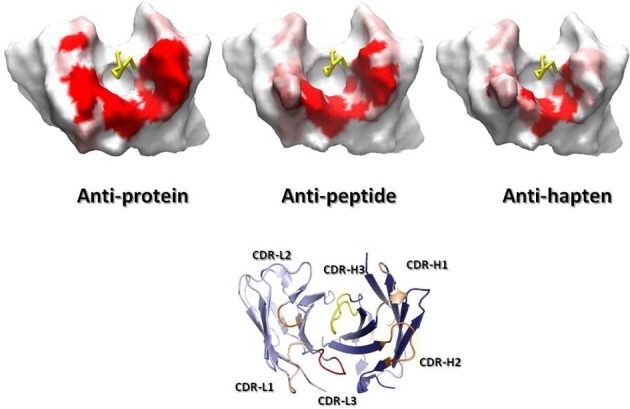
**Connolly (1983) surfaces of representative anti-protein, anti-peptide and anti-hapten Fvs, shown from the antigen perspective.** A gradient from red (contact) to white (no contact) represents the SDRUs of antibodies recognizing generic ligands. The surfaces were generated by running a 7.0-Å radius probe over the Fv after removing the CDR-H3 loop (yellow ribbon) to better represent the surface common to all antibodies. Note the variation in size of the surface from anti-protein, a large surface, to anti-hapten antibodies, a small one. On the bottom, a ribbon representation of a Fv in the same orientation as the Fvs displaying the Connolly surface to indicate the position of the CDRs. Also note the protruding CDR-L1 in the anti-peptide and anti-hapten Fvs, which modulates the topography of the antigen-binding site. The ribbon figure was generated with PyMol, while the Connolly surface figures were produced using Discovery Studio.

Combining the SDRU patterns with the distinctive shape of the antigen-binding site of antibodies recognizing different types of antigens lead to the conclusion that anti-protein antibodies tend to have flatter and larger binding sites than anti-peptide and anti-hapten antibodies. The antigen-binding of anti-peptides is grooved, whereas, anti-hapten antibodies have a smaller and deeper antigen-biding site, with SDRU hotspots buried in the V_H_:V_L_ interface (Figure 5).

Since SDRUs are a measure of the likelihood of establishing contacts with the antigen, they can provide a definition of the antigen-binding site in absence of the antigen-antibody complex structure. A definition of the antigen-binding site based on SDRUs could thus guide the selection of residues to transfer the specificity from a given antibody into a different scaffold, either to produce a molecule with enhanced biophysical profile such as increased stability (Ewert et al., 2004) and/or to humanize a nonhuman antibody (Almagro and Fransson, 2008). Importantly, protocols using SDRUs can tailor humanization of antibodies recognizing different types of ligands, thereby minimizing the region of the non-human antibody grafted into the human context and hence potential immunogenicity.

Not all amino acids are equally used to contact antigens and the types of antibody residues involved in contacts with proteins, peptides, and haptens also differ. Tyrosine (Y), arginine (R), asparagine (N), aspartic acid (D), histidine (H), serine (S), and threonine (T) make more contacts than other amino acids in all the three antigen types. Of particular interest is Y, which has been found in a high proportion in the antigen-binding site of antibodies. For instance, Lo Conte et al. (1999) observed that Y contributed to 16.6% of all amino acids in contact in the 19 antigen-antibody complexes available at that time. Similarly, an earlier report by Mian et al. (1991) based on the analysis of only six antibody–antigen complexes reported the overuse of Y to contact antigens. Cysteine (C), proline (P), glutamine (Q), glutamic acid (E) and hydrophobic amino acids such as alanine (A), valine (V), isoleucine (I), leucine (L), methionine (M) and phenylalanine (F) make significantly fewer contacts. Thus, hydrophilic amino acids predominate over hydrophobic ones. In the CDR-L1, N, and D are the most frequent residues in contacts. R is rare and tryptophan (W) does not occur. CDR-L2 has less diversity than CDR-L1 and CDR-L3, and in the latter, most contacts involve amino acids S, T, W, and Y, whereas, R, N, G, and H make fewer contacts.

A detailed analysis of the contribution of each amino acid called Specificity-Determining Residues Matrix (SDRM) to each SDRU depending upon the type of antigen the antibody interacts with have been described by Ragunathan et al. (2012). Briefly, there are more D and T contacts in anti-protein antibodies in CDR-L1 than anti-peptide and anti-hapten antibodies. In CDR-L3, anti-protein antibodies have more R and W contacts, whereas, anti-hapten antibodies have more Q, G, and H contacts. Similar to V_L_, S, T, and Y dominate the contacts for V_H_. Likewise, C, P, Q, D and hydrophobic amino acids are significantly underrepresented at contact residues. Interestingly, V_H_ has more contacts involving negatively charged amino acids and fewer K residues in comparison to V_L_. In CDR-H1, N, G, S, T, and Y predominant in contact sites for all antibodies. In addition, D occurs frequently in anti-protein and anti-peptide antibodies. The detailed picture of the contribution of each amino acid (SDRM) to each SDRU depending upon the type of antigen the antibody interacts with has practical applications to design antibody repertoires.

## The antibody repertoire in humans and immunization of host species

In addition to the structural studies outlined above, antibody repertoire analyses and comparative immunogenetics have been highly informative approaches to understanding how antibodies evolved to recognize diverse antigen structures. Indeed, the lessons learned from such studies have been critical factors in the progress of antibody engineering. For example, a profound understanding of the biases inherent in the functional repertoire of human antibodies, in comparison to those of other species (Schroeder et al., 1995; Zemlin et al., 2003; Schroeder, 2006) inspired experimental work to define the critical biochemical characteristics required to form a functional synthetic antibody repertoire (Fellouse et al., 2006; Birtalan et al., 2008, 2010). To further illustrate the important influence these studies, below we outline what has been learned about the human antibody repertoire and several other species of interest in antibody discovery and highlight how this knowledge is impacting the antibody engineering field.

### The human antibody repertoire

The primary repertoire of antibodies is produced via the combinatorial rearrangement of IG (H, K, or L)V with IGHD (only in V_H_) and IG (H, K, or L)J germline genes, followed by pairing of V_H_ and V_L_ domains (Tonegawa, 1983). This repertoire should be diverse and versatile enough to recognize any antigen with a low or medium affinity during the primary immune response (Neuberger and Milstein, 1995). The physical maps of the human IGH and IGL gene loci were elucidated in the 1990s (Tomlinson et al., 1992; Schäble et al., 1994; Matsuda et al., 1998) and the information has been compiled and annotated at The ImmunoGenetics Database (IMGT; http://www.imgt.org/). This information has provided the foundations to understand the mechanisms of generation of diversity in human antibodies and has shed light on the evolution of the antibody repertoire.

Overall, the human IGK locus contains approximately 30 functional IGKV genes distributed in six families, and five IGKJ segments which recombine to form the primary Vκ repertoire. There are 30–36 functional IGLV genes arranged in three distinct clusters containing 11 IGLV gene families and four functional Cλ domains, each with its own IGLJ gene. The IGH locus contains approximately 39 functional IGHV genes distributed in seven IGHV gene families, approximately 30 IGHD segments classified also in seven families and six IGHJ genes. As more human germline genes from diverse individuals have been sequenced and studied, an increasing number of alleles have been compiled at IMGT (Lefranc et al., 2005).

Analysis of the antibody genes amplified from diverse sources (Cox et al., 1994; Huang et al., 1996; Ignatovich et al., 1997; Brezinschek et al., 1998; de Wildt et al., 1999; Farner et al., 1999; Glanville et al., 2009) indicates a strong bias in gene usage. For instance, only five IGHV genes (5–51, 1–69, 1–2, 4–59/61, and 3–30/33) make 50% of the rearranged antibodies and only 24 out of 39 functional genes (~60%) are expressed with a frequency above 1% (Glanville et al., 2009). For IGVK the bias is more dramatic. Only three IGVK genes (3–20, 1–39, and 3–15) make 50% of the rearranged antibody repertoire and only 16 out of 30 genes are expressed with a frequency of more than 1%. Pairing of heavy and light chains in B cells (de Wildt et al., 1999) and in recombinant libraries (Glanville et al., 2009) appears to be a random process, reflecting the relative abundance of the IGHV and IGLV gene family members. The bias in the gene usage is due to a number of factors including position in the locus, ontogenetic regulation of the immune response, gene copy and binding properties of the antibodies encoded by certain genes (Dal-Bo et al., 2011; Lerner, 2011; Zhu et al., 2011).

After antibody exposure to antigen, an affinity maturation process generates diversity from which antibodies with higher affinity are selected, as the antigen concentration decreases during the secondary immune response. Affinity maturation mechanisms include somatic hypermutation (in most mammalian systems) and gene conversion (in certain species, see below). The somatic hypermutation process takes place in the germinal centers with the help of T-cells. The V-genes in activated B cells undergo activation-induced (cytidine) deaminase (AID)-catalyzed somatic hypermutation at a rate of up to 10^−3^ changes per base pair per cell cycle (Rajewsky et al., 1987). Two separate mechanisms are involved in the mutation process (Maizels, 2005); one targets mutation hotspots with the RGYW (R = purine, Y = pyrimidine, W = A or T) motif (Dörner et al., 1998) which includes the reverse complement of the preferential substrate site for AID, while the second incorporates an error-prone DNA synthesis that can lead to a nucleotide mismatch between the original template and the mutated DNA strand (Rada et al., 1998). The overall process favors single-base transitions over transversions at a 3:1 ratio (Betz et al., 1993).

The frequency of mutations in V_H_ and V_L_ are qualitatively similar, following an exponential distribution with as much as 15–20% of the V-regions showing no mutations at the amino acid level (Tomlinson et al., 1996; Ramirez-Benitez and Almagro, 2001). The average number of mutations per V-region has been estimated for humans and mice to be around 8 and 5 mutations for V_H_ and V_L_, respectively (Tomlinson et al., 1996; Ramirez-Benitez and Almagro, 2001; Clark et al., 2006). Although the mutations are spread throughout the V-domains, they occur at a proportion of 3:2:1 mutations at the antigen-binding site, surface of the V-domains and V_L_:V_H_ interface, and core of the V-domain, respectively (Clark et al., 2006). The relatively high proportion of mutations in the CDRs with respect to FRs is explained in part by a higher concentration of mutation hotspots in the former. It also reflects the selection for affinity improvement, although it has been found that somatic mutations in residues in direct contact with antigen are less frequent than in residues adjacent to the residues in contact (Ramirez-Benitez and Almagro, 2001), suggesting that the residues selected during the primary immune response do not change during the affinity maturation. Insertions and deletions also occur but at a lower rate (Wilson et al., 1998; Zhao and Lu, 2010), implying that the overall geometry of the antigen-binding site as defined by the canonical structures does not change significantly during the affinity maturation process either.

The amino acid content and length distribution of the CDR-H3 region is of critical importance to antibody repertoire function and the diversity encoded in this loop in humans has been extensively characterized to aid synthetic mimicry of human diversity (Schroeder et al., 1987; Zemlin et al., 2003; Schroeder, 2006; Glanville et al., 2009). These studies have shown very clearly that the human CDR-H3 repertoire is distinctly different from that of the mouse, particularly in length distribution. The human loops tend to be significantly longer, at 15.2 (±4.1) residues, while mice average at only 11.5 (±2.7) (Zemlin et al., 2003). Both species exhibit common conserved motifs at the stem of the loop, but the biases in amino acids used overall and, indeed, in a positional sense, show distinct differences. While humans and mice both show a strong preference for the use of Y, S, and G residues, this phenomenon is much more pronounced in mice (26% Y), than humans (14% Y) and in both species this trend toward high Y use increases proportionally with loop length. In humans in particular, longer CDR-H3 loops are associated with increased use of the IGHJ-6 segment, which encodes a series of contiguous Y residues, increasing the frequency of Y content overall (Prassler et al., 2011; Zhai et al., 2011). In addition, humans use more P and do not exhibit the clear hallmarks of hydrogen bond ladder formation in the loop as often as mice, suggesting more complex overall loop topology in humans. This phenomenon may be directly correlated with increased length in human CDR-H3, with an associated higher use of cysteine via germline-encoded “D2” DH sequences. These long D-segments encode for cysteine residues spaced four amino acids apart, allowing disulphide loop formation that can be critical to CDR secondary structure and rigidity (Almagro et al., 2012). While these disulphide-stabilized loops are relatively rare in humans (C = 1.21% of all amino acid use in human CDR-H3), (Zemlin et al., 2003) they are a commonly used motif in the antibodies of both chickens and camelids, as outlined later.

### Harnessing non-human antibody V-gene repertoires

Species such as mouse, chicken and camelids (such as llama) are all used as immune sources of antibodies with therapeutic potential. While antibodies from human libraries theoretically contain “fully human” amino acid sequence in their FRs, antibodies from immune animal repertoires do not. Nonhuman-derived antibodies may initially have their immunogenicity reduced by cloning the V-genes onto a set of human C regions, to form a “chimeric” antibody (Morrison et al., 1984). Even the small amount of “foreign” amino acid content with respect to humans in the V-domain FRs of a chimeric IgG may be enough to provoke an anti-idiotypic antibody response, however, especially patients that receive repeated doses of antibody as therapy (Stephens et al., 1995). As a result, before clinical use, antibodies derived from animals usually undergo a process of “humanization,” whereby recombinant DNA technology is used to “graft” the CDRs of the clone of interest onto human V-gene framework scaffolds (Jones et al., 1986). It is typically necessary to carry out subsequent V-gene engineering, e.g., via “back mutations” in the FRs, to return the target binding affinity of the parental clone (Almagro and Fransson, 2008). For this humanization process to be efficient, it is helpful not only to be able to predict which human FRs might be optimal to accept the grafted CDRs from a lead clone, but also to understand the nuances of the structural characteristics of the repertoire from which the clone was derived. Armed with sufficient prior knowledge of each species' repertoires, we can confidently predict the likely engineering path that will be required to derive a fully active, but maximally humanized product.

### The mouse antibody repertoire

The mouse (*Mus muscullus*) is the most widely used model organism in immunology and perhaps in biology and medicine. For the study of antibodies, the development of hybridoma technology, first described by Köhler and Milstein (1975) and awarded the Nobel Prize in 1984, was the key advancement that ultimately led to development of antibody-based drugs. Hybridoma technology involves the immunization of rodents with an antigen of interest and once a satisfactory immune response against the antigen has been obtained, the antibody-producing B cells are harvested and fused to a murine myeloma cell line. The resulting hybrid cells can be sub-cloned to generate clonal cell lines in which every cell secretes antibodies with a single specificity. Thus, hybridoma technology became an efficient means to produce unlimited amounts of single-specificity antibodies which enabled the biochemical and structural characterization of antibodies and the production of sufficient quantities of high quality protein for therapeutic settings.

The physical maps of the mouse IGH and IGL gene loci were elucidated in the second half of the 1990s (Tomlinson et al., 1992; Schäble et al., 1994, 1999; Matsuda et al., 1998; Thiebe et al., 1999) and, as for humans, the information is compiled and annotated at IMGT (http://www.imgt.org/). The total number of mouse (*M. musculus)* IGK genes per haploid genome is 164 (174 if the orphons are included), of which 99 are functional, belonging to 18 subgroups (Martinez-Jean et al., 2001). Eighty-one are in opposite orientation of transcription, 59 of them are functional and must rearrange by a mechanism of inversion. These genes are recombined with five IGKJ genes. The IGL locus contains only three IGLV genes each with one associated IGLJ gene. The reduced contribution of the IGL locus to the mouse germline repertoire is consistent with the approximately 8-fold reduction in the prevalence of lambda-bearing IgG in the serum of mice compared to humans.

The IGH locus is both larger and more diverse than that of the humans (Schroeder, 2006). IMGT reported as of August 2012 two tables for the mouse IGVH germline gene repertoire. One with IGHV genes compiled from diverse sources, which represent genes characterized in several strains and thus some genes may be alleles. The other table compiles data from the C57BL/6 Mouse Genome Sequencing and is provisional since not all the genes have been mapped and confirmed. It lists 170 IGVH germline genes distributed in 15 IGHV gene families. One hundred one out of the one hundred seventy known genes (~60%) are functional genes. These IGHV genes recombine with 21 functional IGDH genes assorted in four families and four IGHJ functional genes.

Interestingly enough, comparisons of the canonical structure repertoire encoded in mouse and humans IGHV genes (Almagro et al., 1997; Bono et al., 2004) indicate that the human structural repertoire has two additional classes (1–1 and 1–3). Thus, the human repertoire is more diverse in structural terms than that of mouse. In addition, the canonical structure class 1–2 is more prevalent in mouse (~60%), while in humans the dominant class is 1–3 (~40%) (Almagro et al., 1997). This divergence, together with phylogenetic analysis of the human and mouse IGHV genes (Bono et al., 2004), indicates that most of the sequences in the human and mouse IGHV loci have arisen subsequent to the divergence of the two organisms from their common ancestor. Identifying these differences between human and mouse genes, which are perhaps a reflection of functional and/or structural constraints at work to balance the free diversification of the antibody repertoire in humans and mice (Almagro et al., 1997), could be useful to select the most human-like genes for humanization of mouse antibodies.

### The chicken repertoire

*Gallus gallus*, the domestic chicken, is a classic model for immunological study. Indeed, “B-cell” derives from the term “Bursal cell,” as B-cells were first recognized as products of the Bursa of Fabricius, a cloaca-associated organ that is critical to immune development in birds (Ratcliffe, 2006). The antibody repertoire of chickens has also been extensively characterized in functional isotype content and at the genomic level (Reynaud et al., 1985, 1989; Ratcliffe, 2006). Their immunoglobulin system is distinct from that of humans and mice as they have structural equivalents of mammalian IgM, IgA, and IgG, but not IgE or IgD. IgM is the major isotype expressed on the surface of their B-cells (Ratcliffe, 2006). Additionally, all chicken antibodies use λ isotype light chains, exclusively (Reynaud et al., 1987). Chicken IgG has 4 Cγ domains, however, and is thought to be a structural relative of both mammalian IgG and IgE subclasses (Parvari et al., 1988). Chicken IgG is also found in a “short” form, lacking the CH3 and CH4 regions. Avian IgG is often described as “IgY” as it can be found at high concentration in egg yolk, but it has been proposed that the full-length form should be called IgG and the short form IgY, to aid their differentiation (Ratcliffe, 2006).

The avian V-gene germline repertoire is extremely simple, with single functional V-genes in both the light and heavy chains, that contain unique V_L_-J_L_ and V_H_-D-J_H_ segments (Reynaud et al., 1989, 1991; Parvari et al., 1990). The chicken V_L_ and V_H_ germline domains are highly homologous to stable and soluble human V_λ_ and V_H_3 families (Ewert et al., 2002, 2003), respectively, and this is maintained across the fully mature repertoire (Wu et al., 2011). The uniformity of chicken V-gene FW sequences renders them highly predictable in humanization (Tsurushita et al., 2004; Nishibori et al., 2006). Despite this simple V-gene system (Reynaud et al., 1983; Parvari et al., 1987a,b, 1988; Ratcliffe, 2006), chickens have a broadly adaptable repertoire that generates high affinity antibodies to protein, peptide and hapten antigens (Yamanaka et al., 1996; Finlay et al., 2005, 2006; Nishibori et al., 2006).

The chicken V-gene system is in stark contrast to that found in humans, mice and primates, which all utilize a large set of V-gene sequences that are highly diverse in both sequence and structure (Schroeder et al., 1990; Schroeder, 2006). In chickens, as in rabbits (Weill and Reynaud, 1992), a distinctly different set of diversification mechanisms are used, including gene conversion (Reynaud et al., 1987, 1989). Gene conversion relies on a single template V-gene being diversified via the incorporation of segments from upstream pseudogenes that lack recombination signal sequences. This process is used to diversify both the heavy and light chains, with mutations being introduced into both CDRs and FRs. For the process to be efficient, it relies on high sequence homology between the pseudogene and the germline gene which acts as the acceptor (Ratcliffe, 2006). A recent chicken V_H_ repertoire analysis suggests the requirement for sequence homology between germline and pseudogene leads to a low level of mutagenesis in the FWs, but hypervariability in the CDRs (Wu et al., 2011). Interestingly, this was coupled with strong maintenance of common CDR structural residues that have also been observed in mammals (Rader et al., 2000; Zemlin et al., 2003; Lee et al., 2004), but modulation of residues that affect V_H_–V_L_ interaction (Padlan, 1994) and CDR structure (Foote and Winter, 1992). The chicken V_H_ repertoire therefore adds significant variability at select FW positions to increase structural diversity, e.g., by changing the angle of interaction between the V_H_ and V_L_ domains (Abhinandan and Martin, 2010).

The CDR-H3 repertoire of chickens differs distinctly from that of humans and mice, in both length distribution and amino acid content. Surprisingly, chickens have only 15 functional D-segments, all of which are highly homologous and some (e.g., D9/12/13, plus D4/8/11) are even identical in amino acid sequence (Reynaud et al., 1991). Additionally, reading frame 1 predominates in chickens (Raaphorst et al., 1997), as reading frames 2 and 3 create sequences containing stretches of hydrophobic residues and stop codons, respectively (Reynaud et al., 1991; Weill and Reynaud, 1992). This form of reading frame control appears to be universal and has also been observed (albeit in different reading frames) for; rabbits, sharks, mice, primates, and humans (Raaphorst et al., 1997; Schroeder et al., 1998; Schroeder, 2006). In reading frame 1, chicken D-segments are biased toward the use of G, S, and Y, as observed in all other vertebrate species studied to date (Zemlin et al., 2003; Schroeder, 2006). In contrast to humans and mice however, chicken D-segments obligately contain C, with the consensus sequence G-S- (A/G)-Y-C- (G/C)- (S/W)-X-A- (Y/E) (X = non-conserved) (Reynaud et al., 1991). This limited initial V_H_ CDR3 repertoire is hyper-diversified both by somatic mutation and the insertion of new sequences via gene conversion. These D-like sequences are donated by pseudogenes and may replace the entire D-segment or only a small section, leading to the creation of “mosaic CDRs” (Reynaud et al., 1989, 1991).

Analyses of CDR-H3 amino acid content in the chicken shows very different paratope chemical composition in comparison to humans and mice (Wu et al., 2011). There is a distinct bias toward small amino acids G/S/A/C/T (but not P), while large aromatic and hydrophobic residues are strongly disfavored, including an unusually low representation of Y, the dominant residue in the repertoires of mice and humans (Zemlin et al., 2003). This observation may be important, as synthetic antibody repertoire studies have suggested that Y is a critical amino acid for target binding (Fellouse et al., 2004, 2005, 2006, 2007). Additionally, the chicken CDR3 repertoire has low representation of positively charged residues (K/R). This may be of practical importance, as excess positive charge in the V_H_ CDR3 is associated with polyreactivity (Li et al., 2001) and poor pK profile *in vivo* (Boswell et al., 2010).

The use of C in the CDR-H3 of >50% of all B-cell clones in the chicken repertoire is suggestive that it plays an important functional role. While humans and rhesus make functional CDR-H3 sequences containing a pair of cysteines (Zemlin et al., 2003; Schroeder, 2006), these are found at low frequency in mature human B-cells, and they are very rare in mice (Raaphorst et al., 1997; Zemlin et al., 2003). The high incorporation rate of C in the chicken CDR-H3 is rendered functional by two mechanisms: (1) frequent use of D-D junctions (Reynaud et al., 1991) to create CDR3s with intra-CDR disulphide bridges and (2) insertion of single C residues in the V_H_ CDRs 1 and 2 for inter-CDR disulphide bonding. These covalent bonds between CDRs are structurally analogous to those observed at high frequency in the immunoglobulins of other species such as camelids (Harmsen et al., 2000), sharks (Dooley et al., 2003; Stanfield et al., 2004), cows (Aitken et al., 1997; Sinclair et al., 1997; O'Brien et al., 1999), pigs (Li and Aitken, 2004), and even the duckbilled platypus (Johansson et al., 2002). It seems likely that the increased use of disulphide binding in long CDRs, by several species, may be highly beneficial to stabilize longer loops that have greater sequence diversity, but could suffer from a lack of structural rigidity that leads to an entropic penalty during binding interactions (Wong et al., 2011; Hackel et al., 2010). Mutagenesis studies have shown that these disulphides, in either IgG or single-domain antibodies, are essential for both V-domain stability and binding function (Lee et al., 2006; Fennell et al., 2010; Govaert et al., 2012).

### Beyond standard IGG structures—natural ‘domain antibodies’

**Figure 6:**
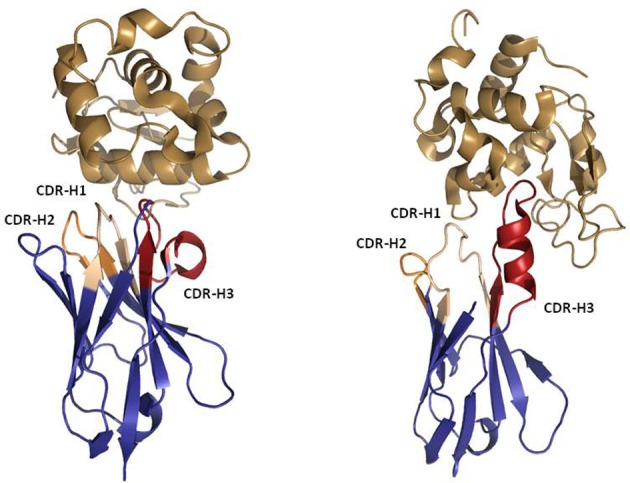
**Ribbon representation of Llama VHH (left) and Shark IgNAR (right) in complex with Hen Egg White Lysozyme (HEL).** PDBID 1OP6 was used to represent the VHH:HEL complex. PDBID 2I26 was used to represent the IgNAR:HEL complex. Note the protruding IgNAR CDR-H3 blocking the active site of HEL, which contrasts with the bended VHH CDR-H3 that recognizes a flat epitope. The Figure was generated using PyMOL.

Despite being the main format for many successful therapeutics, IgG molecules have some practical limitations as they are large (~ 150 kDa), covalently-linked tetrameric structures that classically contain two antigen-binding sites. The necessity for two V-regions to combine and stabilize each other makes it technically challenging to reduce antibodies to anything smaller than the dual-domain single chain Fv (scFv) antibody fragment (~ 30 kDa). The desire for smaller, more stable and monomeric binding modalities in appropriate indications has led to the investigation of a logical alternative; modular therapeutics built from naturally-occurring binding proteins that can be used as a source of “domain antibodies.” As outlined below, comparative immunogenetics led to the discovery of non-classical immune proteins such as the camelid VHH and the shark V_NAR_ (variable domain of the IgNAR), which can both be isolated as soluble, stable, monomeric V-domains (Figure 6) (Flajnik and Dooley, 2009; Wesolowski et al., 2009; Flajnik et al., 2011). These single domain proteins are only ~ 12–15 kDa in size and have been the subject of significant academic and industrial research to characterize their origins and utilities (Muyldermans et al., 2009; Flajnik et al., 2011). Humanization of VHH antibodies is facile, as the isolated antibodies are typically close to a human VH germline sequence. Together with high stability and low aggregation, this gives the humanized VHH antibody theoretically low immunogenicity risk. To date, the less heavily investigated IgNAR has not been extensively characterized in humanization studies and may represent a different challenge from VHH. The VNAR domain is actually more structurally related to a T-cell receptor α-domain and has much lower a.a. identity to human homologous domains. As a result, in this section we concentrate on the more experimentally advanced VHH.

Domain antibodies, lacking an Fc region, suffer from fast renal clearance and without protein engineering they have short *in vivo* half-lives. Luckily, the stable, soluble nature of isolated domain antibodies renders then relatively simple to engineer in a modular fashion. Modifications at the N- or C-terminus are typically possible without loss of function, allowing fusion to common half-life extension molecules such as serum albumin or immunoglobulin Fc and covalent conjugation to natural or non-natural polymers that expand the hydrodynamic radius of the proteins, greatly reducing renal clearance. Domain antibodies have also been extensively exploited as modular units to create bispecific molecules for targeting multiple disease mediators with a single polypeptide (Gill and Damle, 2006; Harmsen and De Haard, 2007). A final exciting avenue open to domain antibodies is the possibility of oral administration, e.g., via strains of *Lactobacillus* expressing a TNF-specific VHH antibody, which was efficacious in a murine gut inflammation model (Vandenbroucke et al., 2010). In the following sections we outline the current state of knowledge surrounding these molecules and the influence that combined repertoire and structural analyses have had on their understanding and application.

### The camel antibody repertoire and VHH

In 1993, the Hamers' group identified a previously unknown immunoglobulin form observed in camel serum (Hamers-Casterman et al., 1993). This new immunoglobulin was found not only to lack a light chain, but to have also deleted the CH1 domain in the heavy chain, following the loss of the splice consensus site (Nguyen et al., 1999). These unique, “heavy chain antibodies” were shown to have a VHH-Hinge-CH2-CH3 structure and performed their antigen binding function exclusively via a stable and soluble VH domain, subsequently dubbed “VHH.” VHH antibodies were found to be fully functional constituents of the immune repertoire of camels, representing >50% of the total Ig population in serum samples (Muyldermans and Lauwereys, 1999; Nguyen et al., 2001). Later studies have shown that other camelids such as llama and alpaca also share these unusual immunoglobulins (Harmsen et al., 2000). The VHH and IgG camel antibodies can be separated by isotype, with IgG1 using architecture of conventional antibodies, whereas the IgG2 and IgG3 isotypes are associated with VHH antibodies (Flajnik et al., 2011).

While they are simple in structure, immunogenetics studies have shown that rather than being a rudimentary evolutionary form of immunoglobulin, the VHH antibody in camelids was actually derived from the genes of a conventional IgH locus by a relatively recent adaptation (Nguyen et al., 2002). Multiple contributory selection pressures have been postulated that might have driven this evolutionary event including; amyloidosis associated with a key light-chain sequence, a virus that targeted a light-chain as a co-receptor, or a simple biophysical pressure to develop high frequency antibodies with a “protruding” CDR structure that is highly appropriate for probing cryptic epitopes (Flajnik et al., 2011). Indeed, multiple co-crystal structures of both VHH (De Genst et al., 2006) and IgNAR (Stanfield et al., 2004, 2007) in complex with enzymes have shown the CDRs to protrude into the active site cleft of the enzyme, neutralizing its function.

Despite relying on a single variable domain for antigen recognition, it has been shown that the VHH repertoire is as complex in sequence diversity as its VH counterpart in camelid IgG1 (De Genst et al., 2006). Indeed, although camelid VHH and VH domains are encoded by distinct sets of V-gene segments, both forms of antibody share some D segments and an identical JH region (Nguyen et al., 2001). Similar to chickens, sequence analysis of camelid VHH domains has shown very close homology to the human V_H_3 family, which is also associated with relatively high stability and solubility (Ewert et al., 2002, 2003). Comparative analyses of VHH and VH germline and repertoire sequences have shown clearly important differences in their respective structures (Riechmann and Muyldermans, 1999; Harmsen et al., 2000), with VHH exhibiting higher frequency of hypermutation hotspots, leading to greater diversity in CDR-H1 and CDR-H2 sequences and length, plus the frequent observation of clones with long CDRs 1 and 3. In another convergence with chickens, VHH antibodies frequently use non-canonical C residues in their CDRs (Govaert et al., 2012). While the disulphide bonding patterns seen in camelids are not as varied as those observed for chickens (Wu et al., 2011), they do lead to disulphide bonding within the CDR-H3, between CDR-H3 and CDR-H1, or between CDR-H3 and FR-2, with the cysteine groups outside the CDR3 typically being placed in very similar positions to those observed in chickens (IMGT positions 38, 55) (Harmsen et al., 2000).

Most critical of all known VHH characteristics, are the hydrophobic to hydrophilic substitutions of four critical residues in the FR-2, known as the “VHH tetrad.” These residues in the FW2 are in critical positions where the V_H_ of a conventional IgG would pack against the V_L_ (Abhinandan and Martin, 2010), providing hydrophobic binding affinity between the two domains. The classic substitutions V37F/Y, G44E, L45R, and W47G lead to a major increase in hydrophilicity of the VHH, allowing it to fold and function independently, without the need for a stabilizing V_L_ partner (Harmsen and De Haard, 2007). This adaptation is essential for biotechnological use, as it allows expression of VHH antibodies at high concentration. While some conventional V_H_ domains can be expressed, they will typically become insoluble at concentrations above 1 mg/ml (Davies and Riechmann, 1994).

Importantly, while long CDR-H3 loops may be common for some VHH sub-types (particularly in camels) and can contribute to solubility by folding over the FR-2, in llama VHH the average CDR-H3 length has been shown to not exceed that observed for humans (Harmsen et al., 2000). Indeed, experimental analyses of independent V_H_ domains derived from chicken IgGs, which do use long CDRs (Wu et al., 2011) but do not contain the FR-2 “tetrad” substitutions, have shown that these domains do not exhibit high solubility and do lose binding affinity when separated from a light chain partner (Finlay et al., unpublished observations). Long CDR-H3 sequences are therefore not a guarantee of FR-2 coverage or solubility in VHH and the FR-2 tetrad appears to be an essential factor in achieving solubility. Studies on the “camelization” of VH domains isolated from monoclonal IgG antibodies have corroborated this, by showing that the addition of the FR-2 tetrad mutations can significantly improve the solubility of those domains (Davies and Riechmann, 1994; Riechmann and Muyldermans, 1999). Nonetheless, camelized and/or CDR-solubilized human antibody domains struggle to replicate the qualities of natural VHH, (Barthelemy et al., 2008) suggesting that combined FR and CDR repertoire content may also play a major role. At the time of writing, no major repertoire analyses have been performed for VHH in the way they have for humans, mice and chickens (Zemlin et al., 2003; Wu et al., 2011).

## Man-made antibody repertoires

Phage display technology was developed by George Smith in 1985 (Smith, 1985) to display peptides on the surface of the filamentous bacteriophage M13. In an effort to isolate “fully human” antibodies and thus bypass humanization, phage display was adapted at the beginning of 1990s (McCafferty et al., 1990) to display antibody V-domain repertoires and to isolate antibodies of interest *in vitro*. During the 1990s and the last decade, several academic laboratories and biotechnology companies have designed and implemented human antibody phage-displayed libraries for antibody discovery (Hoogenboom, 2005; Bradbury, 2010). Such libraries have enabled the isolation of high affinity and specific antibodies against a wide range of molecules and the antibody library design and implementation process continues to evolve.

Since phage display bypasses immunization, it is especially useful for obtaining antibodies against targets that are highly conserved across species and those that may be toxic, where *in vivo* methods are ineffective and/or impractical. In addition, since phage display technology allows access to the repertoire of genes intended for expression and display on the phage surface, the number of genes and variants can be designed or chosen to bias the repertoire toward genes with predefined characteristics, opening up the possibility of testing hypotheses on how the size of a repertoire, its diversity and composition impact the selection of specific, stable, soluble, and high affinity antibodies. Overviews of different man-made repertoires and how their performance has enhanced our knowledge of the evolution of the antibody repertoire are provided below.

### Natural (naïve) repertoires

Originally, human antibody phage-displayed libraries for *in vitro* discovery were implemented either by cloning the natural repertoire of rearranged antibody genes (Marks et al., 1991) or by rearranging human antibody germ-line genes *in vitro* (Griffiths et al., 1994). This first generation of natural or naïve repertoires contained the diversity harvested from the total B-cell repertoire by RT-PCR and thus suffered from a lack of control over FR usage and mutation rate (Sidhu and Fellouse, 2006). This can be a significant issue in antibody therapeutic development, as not all human FRs are used at high frequency in the B-cell repertoire (see above) and, importantly, not all will express well in heterologous systems or be stable in delivery formulations (Ewert et al., 2003). Additionally, antibodies from naïve libraries may contain somatic mutations leading to FR or CDR based T-cell epitopes and/or aggregation-prone sequences that could potentially lead to immunogenicity (Harding et al., 2010). As a result, several research groups have developed fully synthetic antibody repertoires in which a few well-expressed and well-behaved scaffolds are used for the repertoire synthesis and diversity is restricted to the CDRs (Pini et al., 1998; Sidhu et al., 2004).

### Synthetic repertoires

The earliest experimental synthetic antibody repertoire was based on single V_H_ and V_L_ scaffolds, introduced limited diversity into the CDR-H3 alone and was used to generate moderate affinity hits against haptens (Barbas et al., 1992, 1993). This library exploited basic knowledge of antibody structure and diversity by placing random amino acid diversity into the exposed regions of the CDR-H3. Amino acid randomization was made possible by the use of PCR and oligonucleotides containing degenerate DNA sequence in the appropriate CDR-encoding codons. The simplest forms of degenerate codons used are based on random incorporation in the first two bases, followed by restricted incorporation in the third position to either G/T (“NNK” codon) or G/C (“NNS” codon). Both of these schemes predominantly incorporate functional diversity, as they encode for 32 codons total, including only one of the three stop codons (amber) and encoding all 20 amino acids, although not at equal ratio. These codons therefore incorporate a reasonable number of translatable polypeptide sequences, so long as the number of contiguous codons used is not so many that one stop codon would be expected per clone. One complicating factor is the obligate encoding of cysteine by each of these codons, leading to a significant number of clones in which thiol groups are presented unpaired, leading to loop malfunction and reducing the overall functional content of the library.

This very simple method of diversification of key CDR loops was exploited by a series of teams (Griffiths et al., 1993, 1994; Viti et al., 2000; Silacci et al., 2005), who used similar methods to diversify both the CDRs H3 and L3 in a variety of FRs, while maintaining key loop stem residues in the CDR-H3 such as Kabat 93, 94, 101, and 102. Despite the simple nature of these repertoires, they have been highly successful at generating antibodies to proteins, peptides, and haptens. The isolated antibodies have proven utility as immunochemical reagents (Neri et al., 1998) and some have even been applied successfully in therapeutic settings (Neri et al., 1995; Carnemolla et al., 1996; Borsi et al., 1998; Brack et al., 2006; Silacci et al., 2006). This antibody construction style was then progressed by examining the additional benefit of adding synthetic diversity in the CDRs 1 and 2 of V_H_ and exploiting the use of a variety of tailored (A.K.A. “parsimonious”) degenerate codons that encoded a smaller number of amino acids at key positions. This improved the quality of the resulting libraries, so they encoded a larger proportion of functionally folded clones and the avoidance of stop codons allowed the incorporation of greater length diversity in the CDR-H3 (Lee et al., 2004; Sidhu et al., 2004).

Further examination of the amino acid content of the mature human, primate and rodent V-gene repertoires, coupled with expanded structural understanding of antibody-antigen interactions, as outlined above, subsequently created highly defined knowledge of positional amino acid usage in CDRs. These studies strongly suggested that the key amino acids used in making functional contacts with protein antigens were highly biased and the adoption of TRInucleotide, or “TRIM” technology (Virnekas et al., 1994) allowed the first opportunities for this knowledge to be fully exploited. TRIM technology is a method based on classical oligonucleotide synthesis chemistry, but at positions of randomization, mixes of trinucleotide phosphoramidites (A.K.A. trimers) are added instead of a series of single base mixes. Each trimer is a fully synthetic codon and the use of precise combinations of these trimers therefore allows the incorporation of positional bias in amino acid mutagenesis libraries (Virnekas et al., 1994).

The earliest use of TRIM technology in antibody repertoire construction did not exploit its full capability, only introducing fully random amino acid diversity into the CDR-H3 of a single framework pair and selecting the library successfully against a series of haptens (Braunagel and Little, 1997). Subsequent landmark studies, however, took the use of trinucleotides to its logical conclusion and attempted to closely mimic the natural human immune repertoire in synthetic form (Knappik et al., 2000; Rothe et al., 2008; Prassler et al., 2011). Knappik et al. (2000) generated the first iteration of the HuCal^©^ libraries, in which they first recognized that 95% of all human antibody diversity is represented in only seven V_H_ and seven V_L_ germline gene families. This observation inspired them to create a library of V-genes built on consensus FRs derived via alignment of each of these families. Into these FRs they placed double-stranded CDR diversity “cassettes” that had been built using trinucleotides to represent each of the amino acids naturally found at all positions in CDRs H3 and L3. They also included length diversity in the CDR3s and canonical structural determinants for the L3, approximately mimicking the natural biases observed in the human repertoire. In the CDR-H3, the natural dominance of G and Y in the human repertoire was closely reflected, with those two residues making up approximately 15% each, of the encoded residues between Kabat 95 and 100s. All other residues were included at ~4% other than cysteine, which was allowed at only ~1% to allow potential generation of the disulphide-constrained loops that are occasionally observed in the human repertoire. Stem loop biases in both the CDRs H3 and L3 were also closely maintained and limited diversity was introduced at several key positions in the structurally conserved Vκ and Vλ L3 loops. This repertoire design was highly effective in generating nM-affinity clones with specificity for a selection of proteins and peptides (Knappik et al., 2000; Marget et al., 2000).

The methods used by Knappik et al. (2000) have since been elaborated upon in a number of reports. Rothe et al. (2008), reported the construction of the next-generation library HuCal Gold™ in which the design strategy was refined to change the CDR design complexity, the structural format of the library (to Fab fragment) and also to move to “CysDisplay” in which the expressed antibody is tethered to the phage via a disulphide bond with a mutant p3 protein. In this library, the CDR diversity was extended to the CDRs 1 and 2 of both V-domains, in addition to the CDR3s. The CDR cassettes were again based on trinucleotide technology and were built to accurately reflect the canonical structures and diversity found in the families upon which each of the consensus frameworks were built, consciously including structures known to be preferred in the recognition of peptides. The finalized library was found to be of very high practical utility, routinely generating antibody specificities and affinities in the single digit nM range that were useful for both therapeutic and reagent purposes (Jarutat et al., 2006, 2007; Ohara et al., 2006; Prassler et al., 2009). This library design had, however, made a critical concession to optimize its function in *E. coli* expression and phage display technology: the FRs were codon optimized specifically to maximize for *E. coli* periplasmic expression rate. In addition, this function had been aggressively selected for by pre-screening all antibody sequences for periplasmic transport as β–lactamase fusions before inclusion in the final library. This resulted in a high frequency of antibodies being selected that performed very well in prokaryotic expression, but poorly in mammalian cell lines used for industrial production of IgGs. As a result, a third iteration “HuCal Platinum™” has since been made which further updated the design and performance of these synthetic libraries, by optimizing codon use to suit both prokaryotic and eukaryotic expression systems (Prassler et al., 2011). This library also further refined the FR use and CDR content to minimize T-cell epitope content and maximize similarity to the human repertoire, by adding length-dependent positional amino acid bias in the CDR-H3 and by switching some V_H_ gene families to fully germline (e.g., VH3-23). These changes made the library higher performing than the HuCal Gold™library in diversity of hits generated, average affinity and expression rate in mammalian cells. Similar libraries that have recently been generated by separate groups have also strongly supported the broad utility and quality of libraries that naturally mimic the human immune system and that these libraries are flexible in display format as they can be selected by alternative phage display systems such as pIX display (Shi et al., 2010).

The overall HuCal™ story is therefore a clear paradigm and example of the intrinsic attraction of synthetic antibody libraries: they can be designed to add positive attributes and to minimize negatives. While fully human natural cDNA-derived libraries are simple to construct and highly functional, negative attributes such as unwanted FR use, somatic hypermutation in frameworks and potential liability sequences such as aggregation motifs, deamidation sites, N-linked glycosylation motifs, oxidation sensitivities and non-canonical disulphide content cannot be avoided as a rule. The very latest libraries make use of a novel DNA synthesis technology known as Slonomics, which has been shown to be an excellent method for the synthesis of molecular diversity, as it allows the production of precise amino acid/codon biases and low dysfunctional sequence content at any given position (Zhai et al., 2011). Analysis of the first large library generated using this technology strongly supports another benefit of carefully designed synthetic antibody libraries: the removal of segmental linkage within the CDR-H3 that limits paratope structural diversity. While natural CDR-H3 sequences have the benefit of being selected for function in the B-cell, synthetic versions are not generated by IGV-D-IGJ recombination mechanisms and therefore, escape potential limitations on self-reactivity that may be imposed by natural tolerance mechanisms (Zhai et al., 2011). Such synthetic antibody libraries are clearly a mature technology and have become an important section of the armamentarium currently in use for therapeutic human antibody discovery.

### Minimalist repertoires

A series of illuminating studies set out to examine the validity of the suggestion that only certain amino acids, with particular emphasis on Y (see above), are essential in the formation of a functional antibody repertoire (Fellouse et al., 2004, 2005, 2006, 2007; Birtalan et al., 2008, 2010; Fisher et al., 2010). It was postulated that antibody critical contacts were predominantly mediated by Y and that small amino acids played a critical support role in creating conformational flexibility and diversity, especially in the CDR-H3 (Koide and Sidhu, 2009). To examine this hypothesis, large phage libraries of human Fabs on a single FR combination were synthesized containing only four amino acids; Y, A, D, and S in solvent-exposed CDR positions, including CDR-H3 (Fellouse et al., 2004). These libraries were capable of generating high affinity and specific antibodies, including antibodies of 2 nM affinities for VEGF. Remarkably, when the four amino acid code was reduced to only Y and S, highly functional repertoires could still be generated, (Fellouse et al., 2005, 2007) with structural studies subsequently showing that Y was indeed the key residue for making critical contacts with antigen, while serine predominantly provided structural flexibility and “space” to accommodate the bulky Y side-chains (Fellouse et al., 2006).

Importantly, Birtalan et al. (2010) have since progressed these studies and demonstrated that W is the only natural amino acid that can be used as a functional alternative to Y in experimental synthetic antibody libraries based on binary diversity (Birtalan et al., 2010). Indeed, co-crystal structure analysis of an antibody from a W/S-containing repertoire with its target HER2 showed clearly that W is a key determinant of the binding specificity (Fisher et al., 2010). Additionally, these investigators have shown that excess content of highly charged residues such as R is a significant risk factor for the high frequency generation of polyreactive clones (Birtalan et al., 2008, 2010). A subsequent study performed using a set of libraries of synthetic single-domain binding proteins has provided supporting evidence for the idea that minimal diversity can be genuinely functional, but that increased amino acid diversity overall is still the best for high function (Hackel and Wittrup, 2010). Synthetic antibody libraries have, therefore, not only allowed the interrogation of fundamental antibody structure/function relationships, but have illustrated what library design elements are critical to maximal function. Future studies will most likely continue to advance this field to make synthetic diversity as reliable as possible.

### Rational repertoires

Finally, based on the finding that the anatomy of the antigen-binding site determines the propensity to recognize a defined type of generic antigen such as a peptide or a hapten, it has been hypothesized that by biasing an antibody repertoire toward the recognition of predefined antigens the probability of obtaining more specific and higher affinity antibodies may increase. This can be rationalized in terms that, when general purpose repertoires, such as naïve or synthetic repertoires, are used to obtain antibodies to a given target, a vast region of the shape space has to be explored to produce specific antibodies. Provided that only an infinitesimally small fraction of all possible functional antigen-binding sites can be explored by using enrichment technologies, such an exploration should be sparse. Consequently, the probability of selecting specific antibodies of higher affinity should increase in repertoires that have been designed to be focused on predefined regions of the shape space.

**Figure 7:**
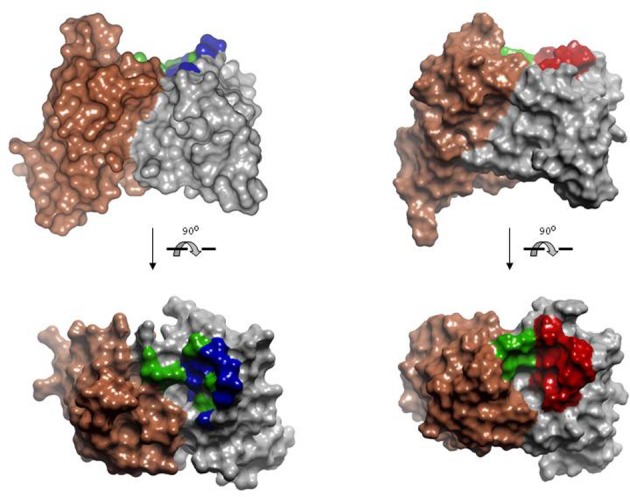
**Model of the human anti-peptide (left) and mouse anti-peptide (right) repertoires.** On the **top**, side view of the Connolly surface of the models. **Bottom**, models seen from the antigen perspective. Invariant V_L_s are colored in brown. V_H_s are colored in gray. Within the antigen-binding site, green represents fully randomized positions, i.e., 20 amino acids; blue positions diversified to Tyr, Asp, Ala, and Ser; red positions diversified to incorporate the most common anti-peptide SDRs. PDBID: 1MCP and 2IGF were used as template for modeling human V_L_ and V_H_, respectively. Coordinates of the antibody 26-10 (PDBID 1IGI) were used for modeling the mouse anti-peptide repertoire. The models and Figures were created in Discovery Studio.

Rational repertoires of antibodies are built by selecting genes encoding combinations of canonical structures that resemble the structural features of antibodies that bind the desired type of ligands. Sequence diversity is then introduced at residues typically involved in recognition of those types of targets. For instance, two antibody repertoires have been designed and tested for peptide recognition (Cobaugh et al., 2008). First, a human anti-peptide repertoire was constructed by pairing the human IGVH germ line gene 3–23 with a variant of the IGVK germline gene 3–20. The CDR-L1 of the gene 3–20 was modified to encode a long loop, typical of anti-peptide antibodies (Figure 7) and diversity was engineered in V_H_ SDRUs of anti-protein and anti-peptide antibodies (see above). Another repertoire was generated using the V-regions of the murine antibody 26–10, which was originally isolated, based on its affinity to the hapten digoxin, but also binds peptides and exhibits a canonical structure pattern typical of anti-peptide antibodies. As in the first repertoire, diversity was introduced in V_H_ only, using the profile of amino acid found at positions that frequently contact peptide antigens. Both repertoires yielded binders to two model peptides, angiotensin and neuropeptide Y, following screening by solution phage panning. The repertoire built onto the 26–10 scaffold yielded antibodies with affinities below 20 nM to both targets.

Another example of a rational repertoire (Persson et al., 2006) was designed using as scaffold the antibody FITC8, which has a cavity in the antigen-binding site, common to anti-hapten antibodies. In five CDRs, diversity was designed on the basis of a 3D model structure of FITC8 and anti-hapten SDRUs. In addition, length variation was introduced into the CDR-H2, as longer versions of this loop have been shown to correlate with increased hapten binding. The repertoire was cloned, phage-displayed and screened against a panel of five haptens, yielding diverse and highly specific binders to four of the selectors. Parallel selections were performed with a repertoire having diversity in more peripherally located residues, which are more often found in contact with protein than haptens. The binders selected from the control (anti-protein) repertoire were not able to bind to the soluble hapten in the absence of the carrier protein, in contrast to the clones selected from the anti-hapten repertoire. Thus, although more validation is needed in order to conclude that rational repertoires increase the probability of obtaining more specific and higher affinity antibodies, these two examples have shown the feasibility and potential advantages of designing repertoires to recognize predefined generic ligands.

## Conclusions and future directions

In this review, we have outlined the importance of the continuum of knowledge that runs through antibody structural studies, species repertoire analyses and experimental exploitation of this information to design antibody repertoires and advance antibody-based drug discovery. Study of hundreds of x-ray crystallography antibody structures free and bound to wide variety of ligands have provided a detailed picture of how antibodies recognize diverse types of ligands with exquisite specificity and high affinity. It has also shown that, although the antigen-binding site is very diverse in sequence and structure, it has predictable geometrical features that determine the types of generic ligands with which the antibody interacts. This knowledge has been critical to understand the mechanisms of the immune response mediated by antibodies and the evolution of the antibody repertoire. Its application has led to the development of engineering methods such as humanization, antigen-affinity optimization and effector function enhancement, which have made possible the approval of more than 30 antibody-based medicines in the last two decades.

The knowledge gained on the antibody structure has been complemented with the study of the antibody repertoire of several species. In addition to humans, we described in previous sections the repertoires and the affinity maturation mechanisms of mice and chickens, plus the use of novel single-domain antibodies in camelids and sharks. These species all utilize diverse evolutionary solutions to generate specific and high affinity antibodies and illustrate the plasticity of natural antibody repertoires. Their comparative study has raised fundamental questions about the evolutionary factors shaping the antibody repertoire such as: has the evolution of the antibody repertoire been a stochastic process or has it been shaped by functional and/or structural constraints? What is the optimal size and diversity of a repertoire that can be generated *in vitro* in order to generate specific and high affinity antibodies to a wide variety of antigen types?

Multiple variations of man-made antibody repertoires have been designed and validated in the last two decades, which have served as tools to explore the above conundrums on how the evolution, size, diversity, and composition of a repertoire impact the selection of more specific and higher affinity antibodies to any given target. A first generation of man-made antibodies included all the antibody genes encoding the repertoire of circulating antibodies, but a relatively limited subset of the genes predominated the panning of the repertoires, showing that many genes are dispensable. A second generation of synthetic repertoires followed. Learning the lesson from the study of natural and man-made naïve repertoires, the new generation of synthetic human antibody repertoires was built on single or a few well-behaved scaffolds. The diversity in these libraries was designed to mimic that of the natural antibodies. These repertoires produced antibodies to a vast array of the diverse antigens. In a further round of design and testing, minimalist repertoires have been designed and validated. These repertoires have been designed to display antigen-binding sites made of very few amino acids or even only binary Y/S and W/S mixes, again yielding antibodies specific against diverse antigens. Finally, rational repertoires encoding genes with predefined recognition features have been tested, which hold the promise of increasing the probability of obtaining more specific and higher affinity antibodies.

Looking forward into the future, new technologies such as next generation sequencing (NGS) are providing the means to study whole natural (Weinstein et al., 2009; Jiang et al., 2011) and man-made repertoires (Glanville et al., 2009) in expedited ways and at relatively low costs (Fischer, 2011; Benichou et al., 2012). Having access to the complete information encoded in repertoires before and after selection under diverse selection pressures, combined with faster and more accurate 3D modeling methods (Almagro et al., 2011; Kuroda et al., 2012) and indeed new conceptual tools and algorithms such as network analysis, may reveal new features of antibody repertoires. These findings will hopefully further impact the theories addressing the origin and evolution of antibody binding specificity. It will also inform the design and optimization of man-made repertoires to isolate more potent, stable, safe and efficacious antibody-based therapeutics, at a lower cost.

### Conflict of interest statement

The authors declare that the research was conducted in the absence of any commercial or financial relationships that could be construed as a potential conflict of interest.
